# Autonomic Nervous System Dysfunction in Primary Sjögren’s Syndrome

**DOI:** 10.3389/fimmu.2021.702505

**Published:** 2021-07-26

**Authors:** Kristen Davies, Wan-Fai Ng

**Affiliations:** ^1^ Translational and Clinical Research Institute, Newcastle University, Newcastle upon Tyne, United Kingdom; ^2^ National Institute for Health Research (NIHR) Newcastle Biomedical Research Centre, Newcastle University and Newcastle upon Tyne Hospitals National Health Service (NHS) Foundation Trust, Newcastle upon Tyne, United Kingdom

**Keywords:** Sjogren, autonomic, fatigue, rheumatoid arthritis, vagus

## Abstract

Primary Sjögren’s syndrome (pSS) is an autoimmune disease which primarily affects the exocrine glands, but can also affect other organs, including the nervous system. Many studies have reported evidence of autonomic nervous system (ANS) dysfunction in pSS which may contribute to a wide range of symptoms and functional burden. Symptoms of ANS dysfunction are common and widespread among patients with pSS and are associated with other features of the disease, particularly fatigue. Accumulating data on the inter-relationship between the ANS and the immune system *via* the vagus nerve have been reported. Vagus nerve stimulation (VNS) has also been associated with improvement in fatigue in patients with pSS. Taken together, these data suggest that the ANS may be a potential treatment target for pSS, in particularly those with fatigue being a predominant symptom. Future research to dissect the link between the ANS, immune dysregulation and clinical manifestations in pSS and to evaluate the potential of VNS as a therapy for pSS is warranted.

## Introduction

Primary Sjögren’s syndrome (pSS) is an autoimmune disease which primarily affects the exocrine glands, although other organs including the nervous system can be affected. The classical features of pSS include oral and ocular dryness, pain, and fatigue (1–3).

Fatigue is often reported as the predominant symptoms experienced by patients with pSS and is a common and disabling symptom of other autoimmune conditions as well as patients with many chronic conditions and cancers (3). Many studies have reported evidence of autonomic nervous system (ANS) dysfunction in pSS (4, 5) and that ANS dysfunction and fatigue are strongly associated (4). Additionally, the presence of ANS dysfunction is associated with a wide range of symptoms and functional burden in pSS (4).

The aetiology of pSS has been largely attributed to immune-mediated mechanisms leading to destruction of salivary and lacrimal glands. However, the degree of exocrine gland destruction in pSS and exocrine function are poorly correlated (6), and objectively measured glandular function does not correlate with subjective report of dryness for some patients (7). Therefore, it has been suggested that the symptoms experienced by patients might not be attributed to the immune process alone (8). Additionally, whilst immune dysregulation provides a plausible molecular basis for the glandular manifestations of pSS, for many pSS patients, the underlying mechanisms of symptoms such as pain and fatigue remain unclear.

Since the function of exocrine glands are highly regulated by the ANS (9), ANS dysfunction may contribute to the clinical manifestations of pSS (8). Interestingly, anti-muscarinic receptor antibodies have been found in saliva and serum of pSS patients (10) and such antibodies have been shown to interfere with muscarinic receptor signalling *in vitro* and reduce saliva production in mice (11). However, it remains unclear whether these autoantibodies play a role in pSS pathogenesis and subsequent autonomic dysfunction. There are extensive “cross-talks” between the ANS and the immune system *via* neural and non-neural communication pathways, with the cholinergic anti-inflammatory pathway among the best described to date (12). Broadly, inflammatory mediators activate the afferent arm of the vagus nerve, which signals are transmitted to the Nucleus Tractus Solitarius in the brain. The efferent arm of the vagus nerve on the spleen and other organs then release acetylcholine from splenic T cells, which in turn mediates anti-inflammatory effects (13). Conversely, sympathetic over-activation can contribute to the initiation and maintenance of inflammation (14).

This article reviews the clinical evidence of ANS dysfunction in pSS and the potential for the ANS as a treatment target in pSS.

## Autonomic Nervous System

The ANS is responsible for maintaining homeostasis of many physiological functions in the body. It consists of the sympathetic and parasympathetic nervous systems, responsible for the “fight-or-flight” and “rest-and-digest” responses, respectively. It is essential for regulating various involuntary functions, such as heart rate, control of respiration, and secretion by glands (15). Here, we review the evidence suggesting ANS dysfunction in various body systems in pSS. A summary of studies on the ANS in pSS is shown in **Table 1**.

**Table 1:** A summary of studies investigating ANS function in patients with pSS.

Authors	Year	Sample Size	Measurement	Main Results
Mandl et al. (16)	1997	19	Reflex tests (DB/FSBP/OC)	Parasympathetic dysfunction
Andonopoulous et al. (17)	1998	32	Reflex tests (DB/SHG/OC/VM)	Autonomic dysfunction
Barendregt et al. (18)	1999	41	Reflex tests	Parasympathetic dysfunction
Niemelä et al. (19)	2000	28	HRV	No difference between pSS and controls
Tumiati et al. (20)	2000	16	HRV	Increased vagal tone
Kovacs et al. (21)	2000	22	CCh induced skin vasodilatation	Impaired vasodilatation
Mandl et al. (22)	2001	30	Reflex tests (DB/FSBP/OC)	Autonomic dysfunction
Barendregt et al. (23)	2002	43	Reflex tests (OC)	Significant differences during OC
Niemelä et al. (24)	2003	30	Reflex tests (DB/OC/VM), HRV	No autonomic dysfunction
Kovacs et al. (25)	2004	51	BPV, HRV	Reduced HRV and BPV
Mandl et al. (26)	2007	46	Reflex tests (DB/FSBP/OC)	Autonomic dysfunction
Cai et al. (27)	2008	27	HRV	Reduced HPV and BPV
Ng et al. (28)	2012	21	Reflex tests (OC/VM)	Autonomic dysfunction
Imrich et al. (29)	2015	21	Reflex tests (OC/IV edrophonium)	Autonomic dysfunction
Koh et al. (6)	2017	154	HRV	Autonomic dysfunction
Brunetta et al. (30)	2019	19	Reflex tests (OC)	Autonomic dysfunction

BPV, blood pressure variability; CCh, Carbachol; DB, Deep breathing; FSBP, finger skin blood flow; HRV, heart rate variability, IV; intravenous OC, orthostatic challenge; SHG, sustained hand grip; VM, Valsalva manoeuvre.

## Cardiovascular Disturbance

The ANS plays a key role in the regulation of the cardiovascular system. ANS dysfunction may result in alterations in the regulation of heart rate (HR), blood pressure (BP), baroreceptor sensitivity, heart rate variability (HRV) and blood pressure variability (BPV). Cardiovascular reflex tests, such as measurements of HR or BP following deep breathing or a Valsalva manoeuvre, changes from lying to standing (tilt table test) can give insight into the autonomic function of the body.

Mandl et al. have conducted several studies exploring the autonomic function in pSS. Their initial study in 1997 comparing 19 pSS patients with 56 age-matched controls showed a significant reduction in systolic BP in pSS patients compared to controls, both at rest and during the tilt table test (16). The impairment of BP response to posture was validated in their follow-up study in 2001, which included 30 pSS patients and 56 age-matched controls (22). Additionally, the investigators reported significant differences in autonomic reflexes such as finger skin blood flow, deep breathing, and orthostatic blood pressure testing in pSS patients compared to healthy controls suggesting dysfunction of both sympathetic and parasympathetic function. They further reported a significant decrease in orthostatic systolic and diastolic BP readings in 46 pSS patients compared to 56 age-matched controls (26). They found similar differences in deep breathing and finger skin blood flow suggesting both parasympathetic and sympathetic dysfunction.

In another study of 51 pSS patients and a historical control group, Kovács et al. reported that whilst pSS patients generally have a normal HR and BP, the majority of patients had a restricted HRV and BPV, as demonstrated by significantly lower median values for HR and diastolic BP in response to multiple cardiovascular reflex tests (25).

Brunetta et al. investigated the cardiovascular autonomic function of 19 patients with pSS by studying elements of the electrocardiogram (ECG), specifically using RR variability alongside systolic pressure variability to create a low-frequency (LF)/high-frequency (HF) ratio (LF/HF) (30). Individually, the LF component is believed to reflect the sympathetic modulation of the sinoatrial node whereas the HF component reflects vagal efferent modulation (6). Thus, the LF/HR ratio represents the relative sympathetic-vagal balance in regulation of the sinoatrial node (31). They found that pSS patients (n=19) had a significantly lower LF and higher HF compared to the control group. The LF/HF ratio was significantly lower in pSS patients compared to controls. Additionally, in response to a head up tilt test, an orthostatic challenge which should increase cardiac sympathetic modulation (31), the increase in the LF/HF ratio was smaller in patients with pSS compared to controls, suggesting a relative impairment of sympathetic function or excessive cardiac vagal modulation (30).

Similarly, Tumiati et al. observed that pSS patients (n=16) had a lower LF/HF ratio in HRV compared to age-matched controls (20). pSS patients also had slower HR and greater R-R variability compared to controls, but these parameters did not reach statistical significance.

Ng et al. investigated autonomic dysfunction by comparing patients with pSS, primary biliary cirrhosis (PBC) and age-matched healthy controls (n=21 for each group) (28). pSS patients were found to have a significantly lower BP compared to controls and had a significant drop in their blood pressure on standing compared to PBC patients. Following a Valsalva manoeuvre, pSS patients reached a significantly lower peak systolic BP compared to controls and PBC patients, where one would expect an overshoot of BP as a normal physiological response (28).

In a study of 27 pSS patients compared to age-and-sex matched controls (27), Cai et al. demonstrated that pSS patients had significantly larger increases in brachial systolic BP and attenuation in the RR ratio in response to standing. Patients with pSS were found to have a relative tachycardia whilst sitting and most pronounced when standing. The authors concluded that pSS patients had reduced HRV and BPV in addition to an increased HR which were more evident in response to a postural change (27).

Koh et al. reported on autonomic dysfunction on 154 pSS patients and age-matched controls (6). They performed a HRV test and found that patients with pSS had a significantly lower HF component compared to controls, but no significant differences in the LF component. Thus, the LF/HF ratio was significantly higher in patients with pSS compared to controls, contrary to the findings from Brunetta (30) and Tumiati (20). A higher LF/HF ratio indicates relatively dominant sympathetic activity

Niemela and colleagues, on the other hand, did not find evidence of cardiovascular autonomic dysfunction studies using 24-hour ECG monitoring and cardiovascular reflex tests (Valsalva manoeuvre, deep breathing test, active orthostatic test, BRS test with phenylephrine) in a group of 30 pSS patients compared to age and gender-matched controls (19, 24).

Thus, conflicting data on objectively measured cardiovascular autonomic function have been reported. The reason for the discrepant data is uncertain, but may include differing methodologies used to measure cardiovascular autonomic function, the inclusion criteria for Sjögren’s syndrome, exclusion criteria for concomitant medical conditions or medication use, the sample size and controls used in these studies. Of note, reduced HRV is a strong and independent predictor of a cardiac event in the general population (32), the HRV test reports better sensitivity and reproducibility compared to reflex tests (33) whilst also correlating well with autonomic dysfunction (34). With this in mind, a long-term follow-up study measuring HRV would be beneficial in exploring the relationship in pSS further (6). Finally, it is possible that autonomic dysfunction affects only subsets of pSS patients.

## Autonomic Dysfunction in Other Organ Systems

Autonomic dysfunction of other organ systems has been reported in pSS. For instance, several studies have demonstrated impaired gastric emptying in patients with pSS (35–37). Gastrointestinal symptoms have been reported more commonly among pSS patients than the general population (37, 38) and it has been suggested that gastroparesis may be underdiagnosed in pSS (37). Since muscarinic receptors are expressed within the gastrointestinal system and autoantibodies directed against these receptors have been detected in pSS patients (35), it is tempting to speculate autonomic dysfunction may contribute to the gastrointestinal symptoms among pSS patients.

Imrich et al. conducted a study of 21 pSS patients (and 13 healthy controls) using edrophonium to systematically assess the parasympathetic cholinergic system (29). Following the administration of edrophonium (an acetylcholinesterase inhibitor which increases the availability of acetylcholine temporarily in the study subjects), a comprehensive battery of assessments of sympathoneural, adrenomedullary, parasympathetic and sympathetic cholinergic function was carried out. They found subtle differences in in several ANS domains but the largest impact was on exocrine function. Four out of 21 pSS patients but none of the controls showed abnormally reduced sweat response, and >50% of pSS patients showed delayed gastric emptying. Interestingly, the impairment of edrophonium-stimulated salivary flows in pSS did not correlate with focus scores or atrophy suggesting that alternative pathogenic mechanisms other than glandular inflammation/destruction may be responsible.

## Autonomic Dysfunction, Disease Activity and Symptomatology in pSS

In addition to studies of objective measurements of autonomic function, several studies have investigated the prevalence of symptoms of autonomic dysfunction in pSS and their relationship with other aspects of the disease. In a large study utilising a subset of 317 patients of the UK Primary Sjögren’s Syndrome Registry (UKPSSR, www.sjogrensregistry.org) (5, 39), autonomic dysfunction was measured using a validated tool, the Composite Autonomic Symptom Scale (COMPASS). COMPASS consists of 6 domains (orthostatic intolerance, vasomotor, secretomotor, gastrointestinal, bladder and pupillomotor), providing an autonomic symptom score ranging from 0 to 100 (40). The study showed that COMPASS scores were significantly higher in pSS patients compared to age-matched controls and that almost 55% of pSS patients scored high enough to suggest autonomic dysfunction. Furthermore, the total COMPASS score correlated independently with ESSPRI (EULAR Sjögren’s Syndrome Patient Reported Index) – a measure of overall symptom burden, and ESSDAI (EULAR Sjögren’s Syndrome Disease Activity Index) – a measure of systemic disease activity. Further univariate analysis was performed but did not show a significant relationship between COMPASS scores and age, disease duration, blood pressure, autoantibody status, sex, erythrocyte sedimentation rate or C-reactive protein. Multivariate analysis demonstrated ESSPRI, ESSDAI and anxiety scores were key independently predictors of COMPASS scores.

Similar findings have been reported elsewhere between COMPASS and ESSPRI (30), ESSDAI (41) and anxiety (42). ESSPRI is made up of three components: fatigue, pain and dryness. Considering the contribution of the secretomotor domain of COMPASS to the COMPASS total score, the correlation between ESSPRI may be expected driven by the dryness domain. Interestingly, pain and fatigue individually were better predictors than dryness for COMPASS total score (5, 41). Tarn et al. recently described the presence of four pSS subtypes with distinct symptomatic and pathobiological profiles, namely ‘Dryness dominant with fatigue’ (DDF), ‘pain dominant with fatigue’ (PDF), ‘low symptom burden’ (LSB), and ‘high symptom burden’ (HSB) (43). It would be of interest to explore whether autonomic dysfunction is more common among the PDF and HSB subgroups.

Although symptoms of autonomic dysfunction correlate with ESSDAI, no such correlation has been reported between ESSDAI and objective measurements of autonomic dysfunction in pSS, which included beat-to-beat haemodynamics and blood pressure measurement in response to orthostasis or Valsalva manoeuvre (28).

Similarly, Mandl et al. reported that whilst symptoms autonomic dysfunction were common in pSS, there were limited association between objective measures of autonomic dysfunction and other clinical features of the disease. Objective measures included in their studies included HRV in response to deep-breathing, blood pressure measurement in response to orthostasis, and finger skin blood flow testing during heating then cooling (26, 38).

Koh et al. studied 154 pSS patients and divided the cohort into those exhibited autonomic dysfunction as measured by HRV (n=55) and those did not (n=99). They found higher prevalence of Raynaud’s phenomenon (p=0.048) and higher ESSPRI fatigue scores (p=0.024) in the autonomic dysfunction group (6). The association of Raynaud’s and autonomic dysfunction is consistent with another study (41).

Taken together, the body of evidence supports the hypothesis that autonomic dysfunction occurs in pSS, but the nature and severity of autonomic dysfunction among individual pSS patients vary. The relationship between autonomic dysfunction, and the pathogenesis and clinical manifestations of pSS remains to be further elucidated.

## ANS as a Potential Therapeutic Target

Several approaches to modulate the ANS have been suggested, which include targeting the baroreceptors, thoracic ganglions, spinal cord and the vagus nerve (12, 44). In rheumatic disease, the target of interest to date has been vagus nerve stimulation (VNS) (45).

Utilising implanted electrodes, VNS has been shown to modulate immune processes *via* the cholinergic anti-inflammatory reflex in both humans and mice (12, 46) and unilateral vagotomy in mice with a knockout of the nicotinic acetylcholine receptor lead to a flare of arthritis (47).

In rheumatoid arthritis (RA), three small studies have shown clinical and biochemical benefit when using VNS (46, 48, 49). The first of these studies was an open-label trial of 18 RA patients unresponsive or intolerant to conventional and biological therapies. VNS lead to improvement in patient-reported measures and in the DAS28‐CRP (28‐joint C‐reactive protein‐based disease activity score) (46). The second was a two-stage pilot study utilising VNS in 14 RA patients which demonstrated significant improvements in clinical and biochemical domains whereas those using a sham device did not (48). The most recent single-arm proof-of-concept study demonstrated a significant reduction in DAS28-CRP in 30 patients with RA refractory to biological therapies over 12 weeks (49). Several small studies which have used VNS have suggested an improvement in symptoms in a wide range of conditions including migraine, anxiety, depression, and fibromyalgia (50–53).

In pSS, the vagus nerve is of particular interest as a potential therapeutic target for several reasons. Firstly, the vagus nerve plays a key role in the communications between the ANS and immune system as mentioned (54, 55). Secondly, observational studies have demonstrated parasympathetic dysfunction in at least in some patients. Thirdly, the vagus nerve is the primary parasympathetic nerve of the ANS controlling essential visceral functions including exocrine glands such as the salivary glands (56). Finally, VNS has been used as a treatment for different conditions with a good safety profile (57).

Indeed, in a study of 15 pSS patients without significant symptoms of anxiety and depression used a non-invasive VNS (nVNS) device twice daily for 28 days, 80% of patients reported improvement in Profile of physical fatigue scores, a validated fatigue assessment tool for pSS (58). Seven participants demonstrated a ≥30% reduction in fatigue within 28 days. A trend of improvement in ESSPRI-fatigue and ESSPR-dryness scores were also observed. Pro-inflammatory cytokine production following ex vivo lipopolysaccharide stimulation of whole blood samples were measured during the study, with IL‐6, IL‐1β, IP‐10, MIP‐1α, and TNFα production significantly reduced over the study period following nVNS (59).

## Discussion

The studies of objectively measured cardiovascular autonomic function yielded inconsistent data, with some showing relative parasympathetic or sympathetic abnormalities while some did not find evidence of autonomic dysfunction. Symptoms of autonomic dysfunction, on the other hand, were more consistently reported in pSS cohorts. The reasons for the discrepant observation may be due to sample size (studies of objective measurement usually have small sample sizes), assessment tools used in the studies, and heterogeneity of the nature of autonomic dysfunction among pSS patients.

The association between fatigue and autonomic dysfunction also deserve more investigations. Fatigue is often described as the most disabling symptom of pSS (3, 60) and has been shown elsewhere to be associated with autonomic dysfunction using multiple measuring tools in pSS (27, 28, 30, 41, 42) and other conditions such as chronic fatigue syndrome and PBC (61). The biological basis for fatigue is still unclear in pSS but immune dysregulation have been suggested (62, 63). In this regard, the existence of cross-talk between the ANS and the immune system raises the possibility that autonomic dysfunction may contribute to fatigue (60).

Considering the lack of approved therapies for pSS (64), VNS represents an attractive potential therapeutic option due to its safety record and much lower costs compared to disease-modifying biologic agents (45). Until recently, VNS require the insertion of an implantable device which may not be acceptable to many patients. The recent development of multiple non-invasive VNS devices provides a safer and more user-friendly option. Early data of using these nVNS devices in RA and pSS showed promise, but the data need to be validated with a larger trial and the inclusion of sham devices to minimise placebo effect. Furthermore, investigation into the optimal strength, frequency, and duration of VNS treatment alongside long-term effects would also need to be determined.

## Conclusion

In summary, symptoms of autonomic dysfunction are common among patients with pSS but with considerable heterogeneity. There is emerging evidence suggesting cross-talk between the ANS and the immune system which may play a role in the pathogenesis of pSS or indeed in the symptoms experienced by patients. New therapies targeting the ANS in rheumatic diseases are promising but remain in early stages. Stratified approach to define the nature of autonomic dysfunction and predictors of therapeutic responses are important.

## Author Contributions

KD and W-FN contributed to the conceptualisation and writing of the manuscript. All authors contributed to the article and approved the submitted version.

## Conflict of Interest

The authors declare that the research was conducted in the absence of any commercial or financial relationships that could be construed as a potential conflict of interest.

## Publisher’s Note

All claims expressed in this article are solely those of the authors and do not necessarily represent those of their affiliated organizations, or those of the publisher, the editors and the reviewers. Any product that may be evaluated in this article, or claim that may be made by its manufacturer, is not guaranteed or endorsed by the publisher.
